# Attitudinal Barriers to Analgesic Use among Patients with Substance Use Disorders

**DOI:** 10.1155/2012/167062

**Published:** 2012-05-03

**Authors:** Leah Zallman, Sonia L. Rubens, Richard Saitz, Jeffrey H. Samet, Christine Lloyd-Travaglini, Jane Liebschutz

**Affiliations:** ^1^Clinical Addiction Research and Education (CARE) Unit, Section of General Internal Medicine, Boston Medical Center and Department of Medicine, Boston University School of Medicine, Boston, MA 02118, USA; ^2^Department of Medicine, Cambridge Health Alliance, Cambridge, MA 02139, USA; ^3^Department of Epidemiology, Boston University School of Public Health, Boston, MA 02118, USA; ^4^Department of Community Health Sciences, Boston University School of Public Health, Boston, MA 02118, USA; ^5^Data Coordinating Center, Boston University School of Public Health, Boston, MA 02118, USA

## Abstract

Attitudinal barriers towards analgesic use among primary care patients with chronic pain and substance use disorders (SUDs) are not well understood. We evaluated the prevalence of moderate to significant attitudinal barriers to analgesic use among 597 primary care patients with chronic pain and current analgesic use with 3 subscales from the Barriers Questionaire II: concern about side effects, fear of addiction, and worry about reporting pain to physicians. Concern about side effects was a greater barrier for those with opioid use disorders (OUDs) and non-opioid SUDs than for those with no SUD (OR (95% CI): 2.30 (1.44–3.68), *P* < 0.001 and 1.64 (1.02–2.65), *P* = 0.041, resp.). Fear of addiction was a greater barrier for those with OUDs as compared to those with non-opioid SUDs (OR (95% CI): 2.12 (1.04–4.30), *P* = 0.038) and no SUD (OR (95% CI): 2.69 (1.44–5.03), *P* = 0.002). Conversely, participants with non-opioid SUDs reported lower levels of worry about reporting pain to physicians than those with no SUD (OR (95% CI): 0.43 (0.24–0.76), *P* = 0.004). Participants with OUDs reported higher levels of worry about reporting pain than those with non-opioid SUDs (OR (95% CI): 1.91 (1.01–3.60), *P* = 0.045). Concerns about side effects and fear of addiction can be barriers to analgesic use, moreso for people with SUDs and OUDs.

## 1. Introduction

Chronic pain is commonly encountered in primary care [1], where the majority of patients with substance use disorders (SUDs) receive health care [2]. Patients with SUDs are at significant risk of pain [3, 4] and are likely to be undertreated for pain. Not only do patients with SUDs report continued pain despite engaging in medical care [5], but few providers caring for these patients follow recommended guidelines [6]. Many pathways may be responsible for such undertreatment, from health systems issues, like insurance coverage and limited access to specialists, to clinician attitudes and skills [7]. For example, clinicians may be appropriately reluctant to prescribe opioid pain medications to patients with histories of substance use disorders out of concern that patients may divert or misuse them [8]. One possible and relatively unexplored reason for undertreatment is patient attitudinal barriers to analgesic medications, such as concerns about side effects, fear of addiction, and worries about reporting pain to physicians. Indeed, patients with SUDs who are HIV positive or are enrolled in methadone maintenance programs report significant attitudinal barriers to pain medications, including concerns regarding addiction potential, need for escalating doses, and difficulty communicating with their clinicians regarding pain [9, 10]. However, such attitudinal barriers to analgesic use, such as opioid medication use, among patients with SUDs in primary care settings have not been well described.

Several patient characteristics have been associated with attitudes that may result in more barriers to the use of analgesic medications (i.e., higher attitudinal barriers) including non-White race, lower education, more physical symptoms [10], older age [11], higher pain severity and disability [12], unemployment [13], and depression [14], while the data on gender are mixed [13, 15]. Other characteristics have not been explored but seem likely to impact attitudinal barriers, such as whether patients are currently or previously addicted to substances, the substances to which they may be addicted (e.g., opioids versus nonopioid drugs), and recent use of analgesics. For example, patients with past addiction, who are currently in recovery, may have more negative attitudes about opioid analgesics than those currently addicted. Similarly, patients with opioid use disorders (OUDs) may be more likely than patients with nonopioid SUDs to have negative attitudes regarding the addiction potential of opioid pain medications. Finally, though psychiatric illnesses, such as depression and anxiety, are known to be associated with more attitudinal barriers [14], the ways in which other psychiatric illnesses, like posttraumatic stress disorder, common among people with chronic pain [16, 17] and SUDs [18], affect attitudinal barriers has not yet been explored.

Our objective was to compare attitudinal barriers to analgesic use among patients with SUDs and those without, across three domains: concern about side effects, fear of addiction, and worries about reporting pain to physicians. We hypothesized that the majority of primary care patients with SUDs would have moderate-to-severe attitudinal barriers and that those with SUDs would have greater barriers in all three domains as compared to those without SUDs. We also explored whether two subcategories of SUDs—(1) current versus lifetime disorder and (2) OUD versus nonopioid SUD—report greater barriers. We hypothesized that people with lifetime SUDs as compared to current SUDs, and OUDs as compared to nonopioid SUDs, would have greater barriers in all three domains. 

## 2. Materials and Methods

### 2.1. Participants and Setting

We conducted a cross-sectional study of a sample of primary care patients with chronic pain. We recruited participants between February 2005 and August 2006 from waiting rooms of an academic, urban hospital primary care practice. We approached patients waiting for appointments and included patients who were 18–60 years of age, spoke English, had pain for three months or more, reported use of any analgesic medication (over-the-counter or prescription) in the prior month, and had a scheduled primary care appointment. Informed consent was obtained from eligible patients. Trained interviewers administered surveys. We compensated participants with $10. The Boston University Medical Center Institutional Review Board approved the study, and a certificate of confidentiality was obtained from the National Institutes of Health.

### 2.2. Instruments

**Dependent Variables**

The dependent variables of interest were the Barriers Questionnaire II (BQ-II) subscale scores. The BQ-II, a validated 27-item self-report survey, was originally designed to measure barriers to obtaining pain relief for cancer patients [11]. We selected the BQ-II because it is a widely used instrument and has been adapted for use with adolescent cancer patients [19] and HIV/AIDS populations [10], and is used in ambulatory settings [10, 19]. We eliminated all questions referring to cancer pain and the immune system in order to make it applicable to a primary care population. The resulting 9-item version, displayed in Table 1, contains three subscales including concern about side effects, fear of addiction, and worries about reporting pain to physicians. The questionnaire refers to “pain medications” and does not specify opioid analgesics. The items are scaled on a 6-point Likert scale from 0 (do not agree) to 5 (agree very much). A-one point increase on the overall BQ-II has previously been shown to be associated with a twofold greater likelihood of being undermedicated for pain control [10]. A BQ subscale score cutoff of ≥3 has been previously used in the literature [10]; we considered a score of ≥3 to be indicative of moderate-to-significant barriers.

**Independent Variables**

The main independent variables of interest were mutually exclusive categories of substance use disorders identified using the Composite International Diagnostic Interview v. 2.1 Drug Use Disorder module, a well-validated interview instrument that yields current and past DSM-IV diagnoses [20, 21].

We assigned participants into three mutually exclusive categories of substance use disorders: those with “OUDs” (which included opioid abuse and dependence), “nonopioid SUDs” (met criteria for a substance use disorder but not an OUD—includes abuse and dependence on alcohol or other drug) and no SUD. If a participant met criteria for more than one substance use disorder and one was opioids, we categorized them as “OUD.”

**Covariates**

We adjusted analyses for factors known to be associated with attitudinal barriers to analgesic use [11, 13–15], or, in the absence of data, due to clinical suspicion that a factor might be associated with attitudinal barriers. These factors were (1) sociodemographic variables including sex, race/ethnicity (Black/African American, Hispanic/Latino/Other, White), employment (unemployed, including receiving disability payments versus employed part or full time), education (<high school, high school+); (2) depression (major or other versus none) determined by the Patient Health Questionnaire (PHQ) for Depression, a 9-item validated measure correlated with past two-week major depression [22]; (3) somatic symptom severity (high versus others) determined by the Patient Health Questionnaire-15, a validated measure that correlates with somatization disorder [23]; (4) a lifetime history of posttraumatic stress disorder (PTSD) diagnosis derived from the CIDI v. 2.1 PTSD module [20]; (5) opioid prescription in the past year. We determined the proportion of participants meeting Prescription Drug Use Disorder (PDUD), defined as any opiate use disorder that was not heroin alone, but did not include PDUD as a covariate in the models because it is a subset of OUD. We determined the proportion of participants with moderate versus severe pain and disability using the Graded Chronic Pain Scale [24], a 10-item validated measure of pain and disability; we did not include pain severity and disability as a covariate in the models because there is little variation in this measure (90% reported severe pain).

### 2.3. Statistical Analysis

**Prevalence of Moderate-to-Significant Barriers**

We compared the proportion of participants with moderate-to-significant barriers in each category of SUD using chi square analyses.

**Current versus Lifetime SUD**

We conducted a sensitivity analysis to examine the relationship between the nonmutually exclusive categories of lifetime (includes both current and past) and current SUDs, and prevalence of moderate-to-significant barriers. Because we did not detect large differences, we used lifetime disorders only for subsequent analyses.

**OUD versus Nonopioid SUD**

We conducted bivariate analyses using general linear models with the Duncan multiple range test for differences to examine the relationship between the independent variable of interest (OUD, nonopioid SUD and no SUD), and the BQ-II subscale scores. Because the three categories differed in their relationship to BQ-II subscale scores (data not shown), we maintained this variable as a three-part variable in further analyses.

**Logistic Regression Analysis of Barriers**

In bivariate analyses, the main independent variable impacted the subscales in opposite directions. We therefore examined the subscales separately. We first conducted bivariate analyses to examine the relationship between covariates and the barrier subscales. We then constructed three multivariable logistic regression models—one for each subscale—in order to examine the relationship between substance use category (OUD, nonopioid SUD, and no SUD) and the odds of having moderate-to-significant barriers. Each model was adjusted for the main independent variable and all covariates. Tests for colinearity among the covariates were carried out using correlation coefficients and any colinear variables were eliminated.

## 3. Results

### 3.1. Participant Characteristics

Of 822 eligible patients, 597 (73%) enrolled and completed the research interview. Enrollees were more likely than those who declined enrollment to be black (61% versus 55%, *P* = 0.04), less likely to take over-the-counter pain medication (66% versus 79%, *P* < 0.001), and more likely to take opioid pain medication (41% versus 30%, *P* = 0.002).

Participant characteristics are shown in Table 2. A majority of the study sample was female, unemployed, and non-white. Roughly half of participants had no SUD, one quarter had an OUD and one quarter had a nonopioid SUD. Of those with an OUD, 104/138 (75%) met criteria for a lifetime prescription drug use disorder. As noted in previous literature [25], the prevalence of OUD is higher among whites (28%) versus blacks (19%) and among males (32%) versus females (17%). 

### 3.2. Attitudinal Barriers

**Prevalence of Moderate-to-Significant Attitudinal Barriers**

In the full sample, moderate-to-significant fear of side effects (60%) and concern about addiction (71%) were common while worry about reporting pain to physicians was relatively uncommon (27%). Participants with OUDs more often reported moderate-to-significant fears of addiction than those with no nonopioid SUDs (89% versus 78%, *P* = 0.02) and no SUDs (89% versus 75%, *P* = 0.01). Participants with nonopioid SUDs less often reported moderate-to-significant worries about reporting pain to physicians than those with no SUDs (18% versus 31%, *P* = 0.01).

**Current Versus Lifetime SUD**

There were no differences in prevalence of moderate-to-significant barriers between those with current and lifetime SUD for any of the subscales (concern about side effects (66% versus 64%), fear of addiction (83% versus 78%), and worries about reporting pain to physicians (23% versus 18%)).

**Unadjusted Analyses**

Unadjusted logistic regression analyses of moderate-to-significant barriers comparing substance use groups are presented in Table 3. Concern about side effects and fear of addiction scores were greater among participants with OUDs than they were for those with no SUDs. Similarly, participants with OUDs had greater fear of addiction than those with nonopioid SUDs. Conversely, scores reflecting worries about reporting pain to physicians were lower for those with nonopioid SUDs than for those with no SUDs.

**Adjusted Analyses**

Multivariate logistic regression analyses of moderate-to-significant attitudinal barrier subscale scores among participants with OUDs, nonopioid SUDs, and no SUD are also shown in Table 3. The greater concerns about side effects and fear of addiction among participants with OUDs compared with those with no SUD persisted in adjusted analyses. Similarly, those with OUDs continued to report higher fears of addiction and higher concern about reporting pain as compared to those with nonopioid SUDs. Differences in worry about reporting pain to physicians between those with nonopioid SUDs remained lower than among those with no SUDs in adjusted analyses. After adjustment, participants with nonopioid SUDs had higher concerns about side effects than those with no SUDs, and participants with OUDs had higher concerns about reporting pain than those with nonopioid SUDs.

## 4. Discussion

In our sample of 597 primary care patients with chronic pain, moderate-to-significant fear about side effects and concern about addiction was common, and more so for participants with OUDs and nonopioid SUDs than those with no SUDs. Conversely, moderate-to-significant worries about reporting pain to physicians were less common overall, and less so for participants with nonopioid SUDs. In adjusted analyses, participants with OUDs reported more concern about side effects and fear of addiction yet similar worries about reporting pain to physicians as those with no SUD. Participants with nonopioid SUDs reported lower worries about reporting pain to physicians than those with no SUD and those with OUDs. Interestingly, those with current and lifetime substance use disorders reported similar barriers with regard to all three subscales.

Why might people with OUDs and chronic pain report a greater degree of attitudinal barriers to analgesic use than participants with nonopioid SUDs? In our study, there were no differences in baseline characteristics between those with OUDs and those with nonopioid SUDs, which suggests that there is something about the experience of the opioid itself (such as the potential addictive quality of opioids) that is associated with increased barriers. Because relapse or worse addiction may be triggered by exposure to opioid medications, those with OUDs may have heightened concern compared to those with nonopioid SUDs, who may perceive less risk of addiction with exposure to opioids. This finding is consistent with other studies showing that HIV-infected patients with a prior injection drug use history are more concerned than their noninjection drug using counterparts about the addictive potential of pain medications [10].

Greater attitudinal barriers among participants with OUDs are particularly interesting because this observation is in contradiction to the experience of many clinicians who describe patients with pain and opioid abuse as “drug seeking” [26]. In fact, while the majority of participants in our study reported attitudinal barriers to analgesic medications, in a study of physicians' perceptions of barriers to AIDS pain management, only a minority of physicians (24%) believed patient reluctance to take opioids to be a barrier to pain management [27]. This discrepancy between physician perception of patient reluctance to take opioids and patient concerns about taking opioids may be a source of miscommunication between patients and physicians. In fact, a clinical encounter for a patient with a substance use history that is focused on pain may have competing demands on the part of both the clinician and the patient. Clinicians balance ambiguity in pain assessment, treatment goals, and treatment risks [28]; similarly, though attitudinal barriers may play a role for the patient, these barriers may not be expressed due to a desire to obtain pain medications for pain relief or misuse, including for their euphoric effect or diversion, all of which are common among patients with prior substance use disorders [5].

The finding that attitudinal barriers were similar for those with current and lifetime opioid use disorders suggests that patients with past SUDs may harbor as many negative attitudes regarding concerns about side effects and fear of addiction potential as patients currently struggling with opioid addiction. This caution about taking pain medications in patients with past SUDs and chronic pain may be appropriate. While opioids may provide good short-term pain relief, chronic opioid use can produce a number of problems, including relapse [29]. However, these perceptions may also lead to undertreatment of pain.

Why might individuals with SUDs have lesser attitudinal barriers to reporting pain to doctors than individuals without SUDs? Patients in this study all suffered from chronic pain and may therefore have developed a certain level of comfort discussing pain. In addition, patients in our study also had ongoing relationships with regular primary care clinicians, a key characteristic identified by patients as essential to good communication and clinical decision making [30]. Finally, patients with SUDs may find it easier to talk about pain than other areas, such as addiction or mental health.

Several limitations apply to our findings. The sample was derived from a single academic urban hospital primary care practice and is therefore potentially subject to idiosyncratic practices, which may limit its generalizability. Nonetheless, the sample reflects a demographically heterogeneous group and is likely similar to patients in many other urban primary care practices in the US. In addition, the questions focused on possible side effects, addiction potential, and reporting of pain, and not about attitudes towards requesting or receiving prescriptions for opioid pain medications. Thus, we may have missed the potentially contentious part of the medical encounter: namely, the dispensation of opioid prescriptions. Finally, the questions did not explicitly delineate which classes of pain medications (e.g., opioid versus nonopioid analgesics) to consider. We expect that most participants assumed questions were referring to opioid medications; indeed recent opioid prescription was common in our sample, suggesting that many participants had experience with prescription opioids.

Despite these limitations, the study offers a preliminary look into the nature of patient attitudinal barriers to analgesic use in primary care patients with pain and addictions. It also offers an alternative perspective on patient-physician interactions than that previously described. Prior studies have demonstrated that the patient-physician interaction regarding pain medications is characterized by mutual mistrust and difficult communication [26]. Primary care providers display discomfort and avoidance in discussing unhealthy alcohol use [31], suggesting that physicians experience difficulty communicating about substance use. However, in this study, patients with SUDs reported low attitudinal barriers to reporting pain to clinicians. They also reported similar concerns to those of physicians, namely, concern regarding pain medication side effects and addiction potential of these medications [10]. These findings may therefore highlight common ground between patients and physicians that offers a basis for patient-physician alliance rather than the previously described “mutual mistrust” [26].

## 5. Conclusions

In summary, patients with opioid use disorders and chronic pain have greater attitudinal barriers to analgesic use than those without opioid use disorders. Patients with substance use disorders have lower attitudinal barriers to reporting pain to physicians. Given the substantial functional, social, and psychological consequences of undertreated pain, there is an imperative for physicians to work with patients who have comorbid pain and substance use disorders to address their attitudinal barriers to pain medications. This study highlights a modifiable barrier to effectively treating chronic pain in patients with SUD. Further clinical care and research should focus on addressing attitudinal barriers so that patients may have maximum pain relief without compromising recovery from addiction.

## Figures and Tables

**Table 1:** Modified Barriers Questionnaire.

Subscale	Item
Side effects	Drowsiness from pain medicine is difficult to control.
	When you use pain medicine, your body becomes used to its effects and pretty soon it will not work anymore.
	Using pain medicine blocks your ability to know if you have any new pain.
	Pain medicine can keep you from knowing what is going on in your body.
	
Addiction	There is a danger of becoming addicted to pain medicine.
	Pain medicine is very addictive.
	
Reporting pain to physicians	It is important to be strong by not talking about pain.
	It is important for the doctor to focus on curing illness, and not waste time controlling pain.
	Doctors might find it annoying to be told about pain.

**Table 2:** Demographic and clinical characteristics of a sample of primary care patients, stratified by substance abuse (*N* = 597).

	Group			*P* value
Variable	OUD	Nonopioid SUD	No SUD	
	*n* = 138 *n* (%)	*n* = 118 *n* (%)	*n* = 341 *n* (%)	
Age, mean in years (SD)	45 (8.9)	45 (8.1)	46 (10.4)	0.66
Race/ethnicity				<0.001^1,2^
Black/African American	70 (51%)	71 (61%)	222 (65%)	
Hispanic/Latino/Other	25 (18%)	23 (20%)	81 (24% )	
White	43 (31%)	23 (20%)	37 (11%)	
Gender				<0.001^1,2^
Female	59 (43%)	54 (46%)	237 (70%)	
Male	79 (57%)	64 (54%)	104 (31%)	
Employment status				<0.001^1,2^
Unemployed or disabled	97 (70%)	83 (70%)	181 (53%)	
Full-/part-time	41 (30%)	35 (30%)	160 (47%)	
Education				0.56
Less than high school	35 (25%)	37 (31%)	94 (28%)	
High school or above	103 (75%)	81 (69%)	247 (72%)	
Depression				0.03^1^
Major and/or other	68 (49%)	54 (46%)	127 (37%)	
None	70 (51%)	64 (54%)	214 (63%)	
Pain severity and disability				0.01^2^
Severe	127 (92%)	113 (96%)	295 (87%)	
Moderate	11 (8%)	5 (4%)	46 (13%)	
Somatic symptom severity				0.06
High	54 (39%)	48 (41%)	104 (31%)	
Low/medium	84 (61%)	70 (59%)	237 (70%)	
PTSD				<0.001^1,2^
Lifetime history	63 (46%)	56 (47%)	100 (29%)	
No history	75 (54%)	62 (53%)	241 (71%)	
Opioid prescription (past year)				0.65
Yes	60 (44%)	46 (40%)	132 (39%)	
No	77 (56%)	68 (60%)	205 (61%)	

^1^Significance <0.05 for comparison between OUD and no SUD.

^2^Significance <0.05 for comparison between nonopioid SUD and no SUD.

**Table 3:** Logistic regression models of moderate-to-significant barriers stratified by substance use disorder^†∗^ (*N* = 597).

	Side Effect		Addiction		Reporting Pain	
	OR (95% CI)	*P* value	OR (95% CI)	*P* value	OR (95% CI)	*P* value
*Unadjusted*						
OUD versus No SUD	**2.09 (1.37, 3.20)**	**<0.001**	**2.78 (1.54, 5.00)**	**<0.001**	0.83 (0.53, 1.29)	0.41
Nonopioid SUD versus no SUD	1.49 (0.97, 2.29)	0.07	1.20 (0.73, 1.97)	0.48	**0.49 (0.29, 0.83)**	**0.01**
OUD versus nonopioid SUD	1.40 (0.83, 2.37)	0.21	**2.32 (1.16, 4.62)**	**0.02**	1.69 (0.93, 3.10)	0.09
*Adjusted*						
OUD versus no SUD	**2.30 (1.44, 3.68)**	**<0.001**	**2.69 (1.44, 5.03)**	**0.002**	0.82 (0.50, 1.35)	0.44
Non opioid SUD versus no SUD	**1.64 (1.02, 2.65)**	**0.04**	1.27 (0.74, 2.19)	0.39	**0.43 (0.24, 0.76)**	**0.004**
OUD versus nonopioid SUD	1.40 (0.81, 2.43)	0.23	**2.12 (1.04, 4.30)**	**0.04**	**1.91 (1.01, 3.60)**	**0.045**

**^†^**Moderate-to-significant barriers defined as score of ≥3 on subscale (range 0–5).

*Models adjusted for gender, employment, depression, somatic symptom severity, education, race, PTSD, and recent opioid use.
